# Formulation of novel niosomal repaglinide chewable tablets using coprocessed excipients: in vitro characterization, optimization and enhanced hypoglycemic activity in rats

**DOI:** 10.1080/10717544.2023.2181747

**Published:** 2023-02-20

**Authors:** Shahinaze A. Fouad, Mahmoud H. Teaima, Mostafa I. Gebril, Fathy I. Abd Allah, Mohamed A. El-Nabarawi, Sammar Fathy Elhabal

**Affiliations:** a Department of Pharmaceutics and Pharmaceutical Technology, Faculty of Pharmacy, Ahram Canadian University, Giza, Egypt; b Department of Pharmaceutics and Industrial Pharmacy, Faculty of Pharmacy, Cairo University, Cairo, Egypt; c Department of Pharmaceutics and Industrial Pharmacy, Faculty of Pharmacy, Badr University in Cairo (BUC), Cairo, Egypt; d Department of Pharmaceutics and Industrial Pharmacy, Faculty of Pharmacy, Al-Azhar University, Cairo, Egypt; e International Center for Bioavailability, Pharmaceutical and Clinical Research, Obour City, Cairo, Egypt; f Department of Pharmaceutics and Industrial Pharmacy, Faculty of Pharmacy, Modern University for Technology and Information (MTI), Mokattam, Cairo, Egypt

**Keywords:** Diabetes mellitus (type II), repaglinide, niosomes, coprocessed excipients, dysphagia, chewable tablets, enhanced hypoglycemic activity

## Abstract

Repaglinide (RPG), a monotherapy insulin secretagogue used to treat diabetes mellitus-type II yet, it suffers from poor water solubility and variable bioavailability (∼ 50%) due to hepatic first pass metabolism. In this study, 2FI I-Optimal statistical design was employed to encapsulate RPG into niosomal formulations using cholesterol,span 60 and peceol^TM^. The optimized niosomal formulation (ONF) showed particle size 306.60 ± 84.00 nm, zeta potential −38.60 ± 1.20 mV, polydispersity index 0.48 ± 0.05 and entrapment efficiency 92.00 ± 2.60%. ONF showed > 65% RPG release that lasted for 3.5 h, and significantly higher sustained release compared to Novonorm® tablets after 6 h (*p* < 0.0001). TEM for ONF showed spherical vesicles with dark core and light-colored lipid bilayer membrane. RPG peaks disappeared in FTIR confirming successful RPG entrapment. To eliminate dysphagia associating conventional oral tablets, chewable tablets loaded with ONF were prepared using coprocessed excipients; Pharmaburst^®^ 500, F-melt^®^ and Prosolv^®^ ODT. Tablets showed friability <1%, hardness 3.9 ± 0.423-4.7 ± 0.410 Kg, thickness 4.1 ± 0.045-4.4 ± 0.017 mm and acceptable weight.All tablets showed robust RPG release at 30 min compared to Novonorm^®^ tablets. At 6h, chewable tablets containing only Pharmaburst^®^ 500 and F-melt^®^ showed sustained and significantly increased RPG release compared to Novonorm^®^ tablets (*p* < 0.05). Pharmaburst^®^ 500 and F-melt^®^ tablets showed rapid in vivo hypoglycemic effect with 5 and 3.5 fold significant reduction in blood glucose compared to Novonorm^®^ tablets (*p* < 0.05) at 30 min. Also, at 6h the same tablets showed 1.5 and 1.3 fold significant extended reduction in blood glucose compared to the same market product (*p* < 0.05). It could be concluded that chewable tablets loaded with RPG ONF represent promising novel oral drug delivery systems for diabetic patients suffering from dysphagia.

## 1. Introduction

Diabetes mellitus (DM) type II is a chronic metabolic disorder that is highly prevalent worldwide. It is the most commonly known type of DM and one of the oldest disorders identified in the world (Olokoba et al., 2012). For clinically diagnosed cases, it accounts for about ninety percent of medical cases (Zimmet, 2003). Moreover, it accounts for being a major cause of morbidity and mortality among human populations (Akash et al., 2011) because of the predisposing complications. DM, type II is characterized by difficulty in carbohydrate and lipid metabolism due to either insulin resistance or impaired insulin secretion or both (DeFronzo, 1999; DeFronzo et al., 2015). Consequently, it is significantly marked by increased blood glucose levels (BGL), i.e. hyperglycemia. Therefore, it was primarily managed using insulin. Although no cure has been emerged yet for this disease, several classes of oral anti-diabetic agents are used in its management either as a mono-therapy or co-administered with insulin. These agents mainly exert hypoglycemic effect (DeFronzo, 1999). They include insulin secretagogues such as; sulfonylureas and non-sulfonylureas as meglitinides, metformin; an oral anti-diabetic agent that subsides glucose production by the liver and insulin sensitizers such as; thiazolidinediones and acarbose; which slows down carbohydrate absorption (DeFronzo et al., 2015).

Repaglinide (RPG) is a short-acting carbamoyl methyl benzoic acid derivative, belonging to meglitinides class members. It is an insulin secretagogue that stabilizes glucose secretion levels during mealtimes (Karami et al., 2020). RPG monotherapy can lower plasma glucose levels in diabetic patients (type II) by stimulating insulin secretion from pancreatic beta cells. Its insulin releasing action is comparable to the first line treatment agents; sulfonylureas and metformin. RPG is a BCS class II drug that suffers from poor water solubility. Therefore, it is rapidly absorbed owing to its high lipophilic nature (log *P* ∼ 3.97). It is also reported for its variable bioavailability ∼ 50%, with well reported hepatic first pass effect (Karami et al., 2020). Moreover, it acquires a very short elimination half-life (t_1/2_) which is nearly one hour (Hatorp et al., 1999; Guardado-Mendoza et al., 2013). Hence, it results in a fast but a short hypoglycemic action which necessitates increased frequency of dosing (DeFronzo, 1999).

Niosomes are nonionic surfactant vesicles that can accommodate active pharmaceutical ingredients (APIs) having widely variable solubility. They have been employed as promising carriers for hydrophilic, lipophilic and amphiphilic drug types. Being nonionic, they are considered nontoxic drug delivery vehicles. They also acquire high chemical stability (Tangri & Khurana, 2011). Niosomes showed their successful applicability to encapsulate various types of active pharmaceutical ingredients (Yadav et al., 2011; Rehab et al., 2021). These include; methotrexate (Azmin et al., 1986), indomethacin (Namdeo et al., 1999) and diclofenac sodium (Naresh et al., 1994). Moreover, niosomes can provide a sustained or extended action for APIs having decreased water solubility and short half-life; as RPG, via encapsulation (Yadav et al., 2011). Extended action allows a consistent form of treatment, especially for chronic diseases such as; DM (type II). As a result, niosomes can provide enhanced patient compliance due to reduced dosing frequency. Several studies showed successful encapsulation of drugs having poor water solubility such as; valsartan (Gurrapu et al., 2012), lornoxicam (Bini et al., 2012), diacerein (Khan et al., 2016) and griseofulvin (Jadon et al., 2009).

Therefore, the aim of the present study favored niosomal encapsulation of RPG; a typical BCS class II API, followed by loading into different types of coprocessed excipients. Coprocessed excipients are directly compressible additives (Gohel & Jogani, 2005) that are employed to develop fast dissolving chewable oral tablets (Moqbel et al., 2016). Chewable tablets offer easy medication access for patients which further improve their adherence to treatment. This is because they readily dissolve in the oral cavity without the need of water for ingestion (Nyamweya et al., 2020) hence, eases oral administration (Dziemidowicz, 2018). For that reason, they are convenient for patients suffering from dysphagia (i.e. difficulty in swallowing) (Hirani et al., 2009) which is prevalent among almost 50% of patients’ population (Malaak et al., 2019). Correspondingly, oral absorption of RPG can be allowed directly from the oral mucosa which will additionally protect the drug from hepatic metabolism. RPG is available in the pharmaceutical Egyptian market as conventional oral tablets; Novonorm^®^, but it is not found as chewable tablets. In our study, developing niosomes as fast dissolving chewable tablets will provide rapid, as well as prolonged effect of RPG to reduce frequency of dosing. Thereafter, assessing the antidiabetic activity of the best selected chewable tablet in rats, in comparison with the market product; Novonorm^®^ tablets.

## 2. Materials and methods

### 2.1. Materials

RPG was kindly supplied from EIPICO, 10^th^ of Ramadan City, Egypt. Glyceryl monooleate (Peceol^TM^) was a gift from Gattefossé, La Défense Cedex, France. Sorbitan monostearate (Span 60) was purchased from Sigma Aldrich Chemical Co., St. Louis, MO, USA. Cholesterol was ordered from HiMedia Laboratories LLC, Pennsylvania, USA. Potassium di-hydrogen phosphate and disodium hydrogen phosphate were bought from El-Nasr Company for Pharmaceuticals, Cairo, Egypt. Prosolv^®^ ODT was supplied from JRS pharma GmbH & Co. KG, Rosenberg, Germany. F-melt^®^ (type ‘C’) was gifted from Fuji Chemical Industry Ltd., Toyama-Pref, Japan. Pharmaburst^®^ 500 was received from SPI pharma, Wilmington, DE, USA. Distilled water was used through the whole study. All other chemicals and solvents were reagent grade and used as received.

### 2.2. Preparation of RPG proniosomes

RPG-loaded proniosomes were prepared according to the method established by Gamal et al. (Gamal et al., 2015). In a stoppered flask, accurately weighed amounts of cholesterol, span 60, and peceol^TM^ were placed and dissolved with RPG (10 mg) in ethyl alcohol (1.5 g). The flask was placed in a warm water bath, adjusted at temperature 65 ^0 ^C ± 2 until a clear dispersion was obtained after nearly 10-15 minutes (min). The aqueous phase (1.5 mL) was then added portion-wise and warmed at the same temperature for 5-10 min until a clear mixture was formed. All mixtures were allowed to cool by continuous mixing at room temperature till proniosomal gels were formed (Sultan et al., 2016).

### 2.3. Preparation of RPG niosomes

In order to obtain RPG niosomes, the previously formed proniosomal gels (section 2.2.) were hydrated by gradual addition of an accurate volume of water with continuous mixing to form 50 mL of the required niosomes. Formulations were left overnight at room temperature to undergo complete hydration. All formulations were subjected to bath sonication for 30 min before in vitro characterization (Sultan et al., 2016).

### 2.4. Formulation optimization

A 2-factor interaction (2FI) I-Optimal statistical design was adopted to evaluate the individual and combined effects of formulation variables using the Design-Expert^®^ 7 software. In the current design, three factors were evaluated; from which two factors were evaluated at two levels and one factor was evaluated at three levels. The studied independent variables were cholesterol concentration (X_1_), Span 60 concentration (X_2_) and peceol^TM^ concentration (X_3_) (Table 1). The selected dependent variables were the particle size (Y_1_: PS), zeta potential (Y_2_: ZP), polydispersity index (Y_3_: PDI) and entrapment efficiency (Y_4_: EE %). Table 2 depicts the composition of the prepared RPG noisomes and the measured responses. Analysis of variance (ANOVA) was executed in order to determine the level of significance (α = 0.05). According to desirability calculations, the optimized formulation was chosen to attain minimized PS (< 800 nm) and PDI (< 0.5) and maximized ZP (> 25 mV; as an absolute value) and EE% (> 50%). Then, the optimized formulation was prepared and evaluated.

**Table 1.** Independent and dependent variables used to optimize RPG niosomes formulations by 2FI I-Optimal statistical design.

Factors (independent variables)	Levels		
	Low (−1)	Medium (0)	High (+1)
X_1_: Cholesterol %	1	–	2
X_2_: Span 60 %	1	3	5
X_3_: Peceol^TM^ %	1	–	2
Dependent variables	Goals		
Y_1_: Particle size (nm)	Minimize		
Y_2_: Zeta potential (mV)	Maximize		
Y_3_: Polydispersity index	Minimize		
Y_4_: Entrapment efficiency (%)	Maximize		

**Table 2.** Experimental runs, formulation variables and measured responses of the 2FI I-Optimal statistical design.

Runs	Independent variables			Responses			
	X_1_: Cholesterol concentration (%)	X_2_: Span 60 concentration (%)	X_3_: Peceol^TM^ concentration (%)	Y_1_: PS (nm)	**Y_2_: ZP** **(mV)**	Y_3_: PDI	Y_4_: EE (%)
F1	2	5	1	1436.0 ± 59	−39.8 ± 2.30	1.000 ± 0.01	64.0 ± 5.20
F2	1	5	1	1258.0 ± 22	−33.7 ± 1.10	0.975 ± 0.01	92.0 ± 1.30
F3	2	1	2	1295.0 ± 44	−30.9 ± 0.90	0.822 ± 0.01	99.0 ± 0.50
F4	1	3	2	277.7 ± 2.6	−32.7 ± 0.70	0.410 ± 0.06	94.5 ± 2.10
F5	2	1	1	1360.0 ± 50	−32.0 ± 0.60	0.852 ± 0.04	91.0 ± 1.60
F6	1	1	1	320.2 ± 3.3	−27.0 ± 1.06	0.323 ± 0.01	97.0 ± 2.30
F7	2	5	2	303.2 ± 2.1	−36.5 ± 0.05	0.485 ± 0.02	87.0 ± 6.70
F8	2	3	2	595.3 ± 4.6	−32.0 ± 0.40	0.767 ± 0.06	92.0 ± 5.40

Each formulation contained 10 mg RPG; Data are mean values (*n* = 3 ± S.D.).

### 2.5. In vitro characterization of RPG niosomes

#### 2.5.1. Determination of niosomes size, physical stability and size distribution

Mean values of particle size (PS), zeta potential (ZP) and polydispersity index (PDI) of RPG noisomes were determined via the dynamic light scattering method using Zetasizer (Malvern Instrument Ltd., Worcestershire, England) at 25 °C. Prior to measurements, all formulations were diluted properly in order to assure that dispersions are translucent and have suitable scattering intensity (Scognamiglio et al., 2013). In order to evaluate the physical stability of formulations and the particle size distribution, ZP and PDI were measured, respectively. All determinations were done in triplicate (*n* = 3).

#### 2.5.2. Entrapment efficiency (EE %)

Entrapment efficiency (EE %) was determined experimentally employing the dialysis method, via cellulose tubing (Trotta et al., 2002). Five mL of each of the prepared niosomes was filled inside a dialysis bag having a molecular weight cutoff (MWCO) 12,000 Daltons (Da) (Sigma diagnostics, St. Louis, MO, USA). Each dialysis bag was tied from both ends and suspended in 100 mL phosphate buffer saline (pH 6.8) for 4 hours (h) at 37 °C ± 0.5 (Abd-El-Azim et al., 2018). The concentration of RPG in the dialysate was determined spectrophotometrically (Shimadzu, model UV-1601 PC, Kyoto, Japan) at the maximum wavelength of RPG (241 nm) (Gadadare et al., 2015). Drug-free niosomes in dialysis bag, treated similarly to RPG-niosomes, provided blank readings at 241 nm.

Percentage of entrapped drug was calculated using the following equation:

$$EE\;\text{\%}=\frac{Ct-Cf}{Ct}\text{*}100$$

(Mehta et al., 2011)where, C_t_ represents the total amount of RPG present in 5 mL noisomes formulation and C_f_ represents the amount of free, dialyzed RPG. All experiments were done in triplicate (*n* = 3).

#### 2.5.3. In vitro drug release of the optimized RPG niosomes formulation

In vitro release profiles of RPG from the optimized niosomes compared to the commercially available Novonorm^®^ tablets (2 mg) were determined. In vitro release studies were determined employing the dialysis bag method having MWCO; 12,000 Da (Sigma diagnostics, St. Louis, MO, USA) (Schlich et al., 2020; Yaghoobian et al., 2020). An accurately measured volume (2.5 mL) of the optimized drug-loaded RPG noisomes formulation (equivalent to 0.5 mg RPG) was introduced into a dialysis bag which was carefully tied from both sides then, carefully tied to the paddle of U.S.P dissolution tester (Apparatus II) filled with 150 mL of simulated gastric fluid (SGF) pH = 1.2 and adjusted at 37 °C ± 0.5. Paddles were rotated at 50 revolutions per minute (r.p.m). Two mL samples were withdrawn at specified time intervals (5, 10, 15, 30, 60, 120, 180, 240, 300 and 360 min) and continuously replaced with equal volumes of fresh dissolution medium to maintain sink conditions. All samples were assayed spectrophotometrically at RPG λ_max_ (241 nm). The same procedures were repeated for the optimized formulation while using the simulated intestinal fluid (SIF) (pH = 6.8) as the dissolution medium. All experiments were performed in triplicate (*n* = 3).

#### 2.5.4. Morphology of the optimized niosomes formulation

Morphology of the optimized niosomes vesicles was imaged via transmission electron microscope (TEM) (JEOL, JEM-1230, Tokyo, Japan) at 120 Kilo Volt (KV). The optimized formulation was prepared by placing diluted drops of the optimized noisomes dispersion on a carbon coated copper grid, stained with 1% phospho-tungstic acid then, it was allowed to dry at room temperature for 30 min. Then, the sample was finally examined under the microscope (Fouad et al., 2018).

#### 2.5.5. Fourier transform infrared spectroscopy (FTIR)

Compatibility between RPG and niosomes components within the optimized formulation including; cholesterol, span 60 and peceol^TM^ was determined via FTIR (FTIR-8400S, Shimadzu, Japan). Each sample (5 mg) was mixed with potassium bromide (100 mg) and compacted into disks by a hydraulic press then, scanned in the range from 400 to 4000 cm^−1^. At the end of the experiments, FTIR spectra of the optimized RPG-loaded noisomes was compared to the corresponding plain niosomes.

### 2.6. Preparation of RPG niosomes-loaded chewable tablets

The optimized RPG niosomes formulation was chosen to prepare RPG containing tablets. Tablets were prepared employing the direct compression technique using a single punch tablet machine under a constant pressure using flat-faced 8 mm punch and die set. Three types of directly compressible excipients were used namely; Prosolv^®^ ODT, Pharmaburst^®^ 500 and F-melt^®^. A suitable volume (2.5 mL) of the optimized noisomes formulation containing an equivalent of 0.5 mg RPG was centrifuged using a cooling centrifuge (Model 8880, Centurion Scientific Ltd., W. Sussex, UK) adjusted at 10,000 rpm for 30 min at −4 °C in order to separate the niosomes from the free (un-entrapped) drug. The supernatant containing the free drug was removed leaving the solid niosomal residue. The obtained residue was left to dry completely for 24 h, then mixed thoroughly with an accurately weighed amount (180 mg) of each directly compressible excipient. The mixture powder of each tablet was fed manually into the die then, compressed into tablets.

### 2.7. Evaluation of RPG chewable tablets

#### 2.7.1. Physical characterization

RPG tablets were physically evaluated via performing tests for weight uniformity, thickness (diameter), hardness and friability. Tests were performed in triplicate (*n* = 3) and according to compendial specifications (Pharmacopoeia, 2002).

#### 2.7.2. In vitro dissolution studies

Dissolution profiles of RPG from the prepared tablets, compared to the market oral tablets (Novonorm^®^, 2 mg) were determined using U.S.P dissolution tester (Apparatus II) adjusted at 37 °C ± 0.5 and 50 r.p.m. The used dissolution mediums were 900 mL SGF (pH 1.2) or 900 mL of SIF (pH 6.8). Three mL samples were withdrawn at specified time intervals (5, 10, 15, 30, 60, 120, 180, 240 and 360 min) and directly replaced with equal volumes of fresh dissolution mediums in order to retain a constant total volume. All samples were filtered through 0.22 μm membrane filter, properly diluted then, measured spectrophotometrically at the predetermined RPG λ_max_. All experiments were done in triplicate (*n* = 3).

### 2.8. In vivo hypoglycemic study

#### 2.8.1. Experimental design

Thirty adult male albino wistar rats weighing 200 g ± 50 were employed in this study. Experimental animals were purchased from Helwan’s Farm (Cairo, Egypt). The in vivo study protocol was reviewed and approved by the Research Ethics Committee (REC) for experimental and clinical studies at Faculty of Pharmacy, Cairo University, Cairo, Egypt (the serial number of the experimental protocol is PI: 2726). Experimental rats were randomly divided into four groups, six rats each. The first group comprised the un-treated, streptozotocin-induced diabetic rats. The second group represented streptozotocin-induced diabetic rats, treated with the market product (Novonorm^®^ tablets, 2 mg). The third and the fourth groups included streptozotocin-induced diabetic rats that were treated with the optimized RPG containing Pharmaburst^®^ 500 and F-melt^®^ chewable tablets per oral (P.O.) administration, respectively. In all experimental groups, the dose was 2 mg/kg and each tablet was dissolved in saline prior to oral administration using a feeding syringe.

#### 2.8.2. Induction of DM (type II)

All rats were allowed to fast overnight. Type II DM was induced in all experimental groups, using single dose of streptozotocin (50 mg/kg body weight) via intra-peritoneal (IP) injection (Hasan et al., 2013). Prior to rats’ injection, streptozotocin was dissolved in 0.1 M citrate buffer (pH 4.5). Diabetic rats were then freely supplied with food and 5% glucose solution in order to prevent sudden hypoglycemia associated with streptozotocin injection (Arafa et al., 2017).

#### 2.8.3. Determination of the hypoglycemic effect of RPG

BGL were checked via using a glucometer (Accu-Chek Aviva meter, USA). Diabetic rats were anesthetized using ether for two min (Akbarzadeh et al., 2007). 0.5 mL of blood was taken from the tail vein (Arafa et al., 2017) and BGL were determined directly before treatment (i.e. at zero time) and at predetermined time intervals after treatments’ administration at 0.25, 0.5, 1, 2, 4 and up to 6 h. Determined BGL for each sample was plotted versus time in order to generate the blood glucose profile for each experimental group.

### 2.9. Statistical analysis

The obtained data for the optimized RPG chewable tablets compared to that obtained from the market product were analyzed for statistical significance by the one-way ANOVA adopting SPSS statistics program, version 16, SPSS Inc. (Chicago, USA). ANOVA was then followed by post hoc multiple comparisons employing the least square difference. Probability, *p*-values less than or equal to 0.05 were considered statistically significant.

## 3. Results and discussion

### 3.1. Statistical analysis and formulation optimization

The 2-FI I-Optimal statistical design was employed in preparation of niosomes using Design Expert^®^ version 13.0.3.0; Stat-Ease, Inc., Minneapolis, MN. This design was applied to get an optimized niosomal formulation with minimized values of PS and PDI, as well as maximized values of EE% and ZP (Table 1).

The individual and the interactive effects of independent variables were investigated (Table 2). The regression analysis and the polynomial equation for each response (Y_1_, Y_2_, Y_3_ and Y_4_) were obtained. A positive sign of the regression coefficient indicated a positive effect, while a negative sign indicated the opposite (AbuElfadl et al., 2021). ANOVA test was applied to detect significance at *p*-value ≤ 0.05. Three-dimensional (3D) surface plots were achieved to perceive the significant effect of independent variables on formulation responses and to select the optimum level for each variable.

### 3.2. PS, PDI and ZP

**Figure 1.**
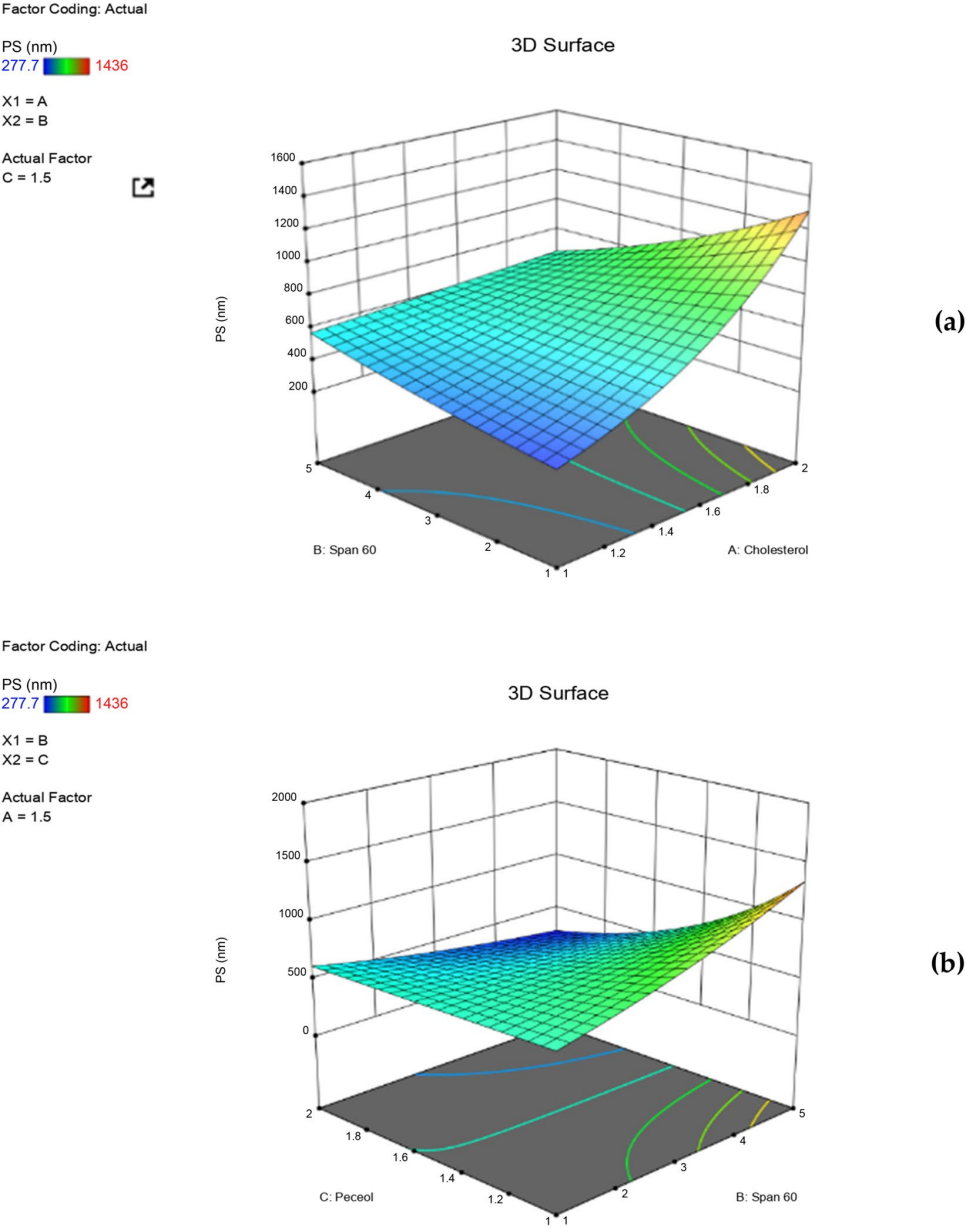
Three dimensional (3D) surface plots for the effect of independent variables on PS.

According to the design of experiments (DoE), lower values of PS and PDI, as well as higher values of ZP were required. Small PS values results in enhanced oral drug absorption and bioavailability (AbuElfadl et al., 2021). Table 2 shows PS results of the prepared RPG-loaded niosomes. Results are expressed as ‘Z- average’ diameter which reflects the average hydrodynamic diameter of the measured vesicles. The mean PS ranged from 277.7 nm ± 2.6 to 1436.0 nm ± 59. The polynomial regression equation of the mean PS was obtained in terms of coded factors, as shown below:

Mean PS=3.35635+ 1.76740 X1+1.04713 X2+0.295733 X3+0.328489 X1X2+0.007384 X1X3−0.376563 X2X3

where, the adjusted R^2^ is equal to 0.9968 showing the good correlation between the independent variables. Large coefficient values indicates the potent influence on the measured response and vice versa. ANOVA results showed that cholesterol had a statistically significant impact on the PS of niosomes (*p* = 0.0257). PS significantly increases upon increasing cholesterol concentration, as clearly demonstrated in the three dimensional (3D) surface plot (Figure 1a). This is because the size of vesicles relies primarily on the molecules present in their bilayers (Balakrishnan et al., 2009), where a highly rigid structure of niosomes is developed upon increasing the cholesterol content with a consequent decrease in particles’ fluidity. In addition, the PS is expected to increase due to the competition between RPG and cholesterol within the bi-layer packing space of the niosomal particles. Similar findings were obtained by AbuElfadl et al. in their study on formation of candesartan cilexetil niosomes (AbuElfadl et al., 2021). Also, increased span 60 concentration led to increased PS, however non-significant (*p* > 0.05). Increase in PS is often directly paired with the length of alkyl chain of the used lipophilic surfactant. This can explain the larger PS of niosomal vesicles accompanied with increased span 60 concentrations where, span 60 acquire a long alkyl chain with eighteen carbon atoms (C18) within its molecular structure. Similar results were achieved by Ning et al. in their study on insulin (Ning et al., 2005) and Balak et al. in their study on minoxidil niosomes (Balakrishnan et al., 2009). ANOVA results also revealed that the increased concentration of peceol^TM^ was found to significantly increase PS of the measured particles (*p* = 0.0247) (Figure 1b). This could be attributed to its molecular structure. Peceol^TM^ acquires bent hydrocarbon chains within its molecular structure, due to the presence of double bonds inside its oleate moiety. As a result, these chains will need more space to occupy within the bilayer structure of niosomes ending in membrane expansion and PS increment (AbuElfadl et al., 2021). Moreover, ANOVA results revealed a statistically significant impact of 2FI of X_1_X_2_ (*p* = 0.0405) and (X_1_X_3_) (*p* = 0.0353) on PS. These findings revealed the synergistic effect of increased concentrations of both cholesterol and span 60, as well as cholesterol and peceol^TM^ on increasing PS values. These results correlate well with the aforementioned effect of each independent variable on the measured PS where, increased rigidness of the niosomal bilayers primarily due to high cholesterol content would develop niosomes that are resistant to size reduction by sonication.

PDI values are dimensionless numerical figures that define homogeneity of PS distribution of vesicles under investigation. In our study, PDI values ranged from 0.485 ± 0.02 to 1.000 ± 0.01 (Table 2). The resulted polynomial regression equation of mean PDI values, obtained in terms of coded factors (adjusted R^2^ = 0.9910) is shown below:

Mean PDI=0.7191+0.0896 X1+0.0288 X2+0.1184 X3−0.0760 X1X2+0.0011 X1X3−0.1212 X2X3

**Figure 2.**
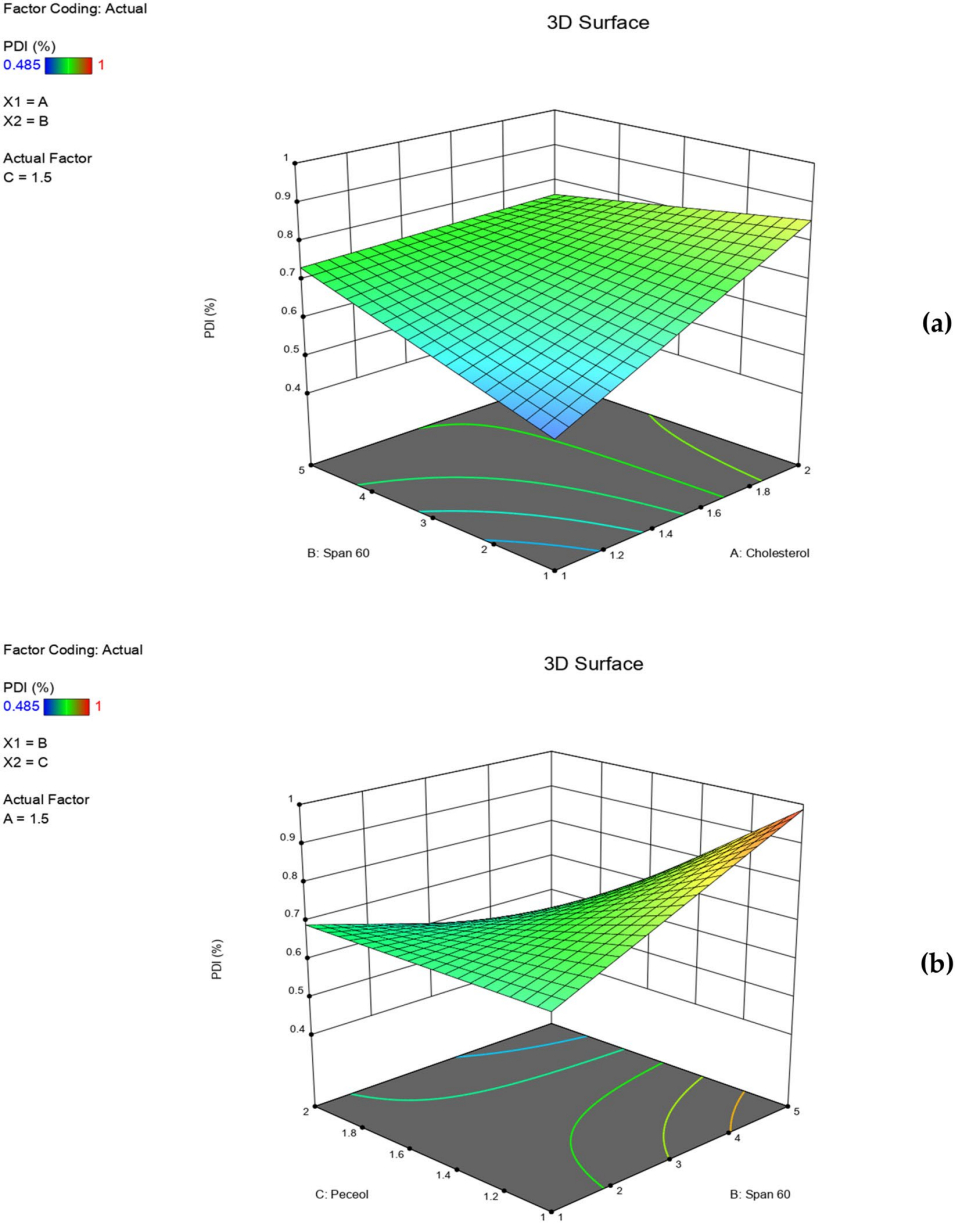
Three dimensional (3D) surface plots for the effect of independent variables on PDI.

ANOVA results revealed that neither the independent variables nor their interactions had a significant effect on the PDI (*p* > 0.05). It was observed that an increase in the PS was accompanied by a parallel increase in PDI values (Table 2). Despite being statistically insignificant, increased cholesterol concentrations led to higher PDI values of niosomes formulations (Figure 2a). As previously mentioned, cholesterol imparts rigidity to the bilayer membranes of niosomes which consequently reduces their fluidity and oppose their size reduction during sonication thus, produces bigger and heterogenous vesicles. Thereby, high PDI values were obtained. Similar findings were obtained by Essa in a study on sorbitan monopalmitate niosomes (Essa, 2010) and Aziz et al. in their study on Diacerein loaded niosomes (Aziz et al., 2018). Highly obtained PDI values could be also attributed to the long saturated alkyl chain of span 60 molecules (Figure 2a,b). As well, the bent hydrocarbon chains in the oleate moiety in peceol^TM^, resulted in enlarged vesicles with heterogeneous PS distribution and hence, high PDI values (Figure 2b).

ZP is a measure of the overall charge attained by the vesicles. It can be used to evaluate the stability of the prepared niosomal formulations. Dispersion systems are considered stable when their ZP values are above ±25 mV where, stability is due to electrical repulsion between the particles (Fouad et al., 2018). The resulting polynomial equation in terms of coded factors (R^2^ = 0.9989) was:

ZP =−33.05+0.1542 X1+4.6 X2+0.2292 X3−1.83 X1X2−0.2708 X1X3+3.62 X2X3

**Figure 3.**
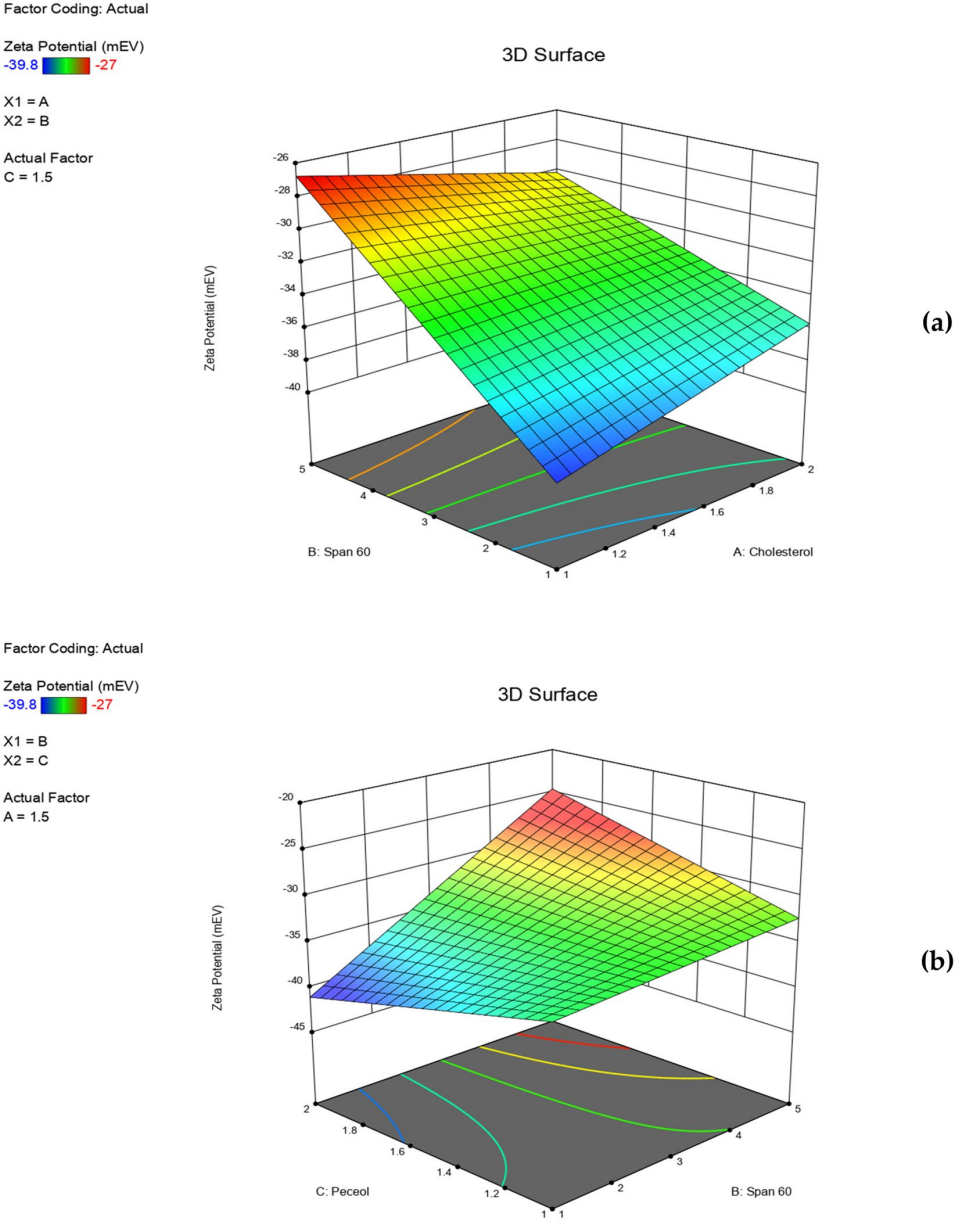
Three dimensional (3D) surface plots for the effect of independent variables on ZP.

ANOVA results revealed that ZP of niosomal formulations were not significantly impacted by any of the formulation variables or their interactions (*p* > 0.05). Results showed that all niosomes formulations had accepted ZP values ranging from −27.0 ± 1.06 to −39.8 ± 2.30 mV. Hence, the prepared RPG niosomes acquired sufficient surface charges that prevented their aggregation and maintained their good stability. In our study, the imparted surface negative charges may be due to several factors. These include incorporation of cholesterol, having hydroxyl moieties that can impart negative charges (Taskinen et al., 2005). Also, span 60 which is a nonionic surfactant that contains hydroxyl ions that can localize at the surface of the niosomes’ membrane bilayer (Soliman et al., 2016). Despite being statistically insignificant, the 3D surface plot (Figure 3a,b) correlated well with the interpreted results where, increased cholesterol and span 60 concentrations led to increased ZP values. In addition, peceol^TM^ could participate in imparting a negative charge and hence increasing ZP values (Figure 3b), due to the presence of oleic fatty acid residuals having ionized carboxylic groups within its molecular structure (AbuElfadl et al., 2021). Moreover, RPG molecules contain ionized carboxylic acid moieties that can be adsorbed on the niosomes’ surface imparting additional surface negative charges (Nicolescu et al., 2010).

### 3.3. EE %

Entrapment efficiency is one of the extremely important parameters in evaluation of niosomal formulations. For that reason, researchers always seek to achieve high EE% values (Balakrishnan et al., 2009). It is overseen by its ability to retain drug molecules either in the aqueous core or within the bilayer membrane of the formed vesicles (Soliman et al., 2016). In our study, EE % of the prepared niosomes ranged from 64.0 ± 5.20 to 99.0 ± 0.50% (Table 2). ANOVA analysis revealed that EE% was significantly influenced by X_1_ (*p* = 0.05), X_1_X_2_ (*p* = 0.0358) and X_2_X_3_ (*p* = 0.0472). The resulting polynomial equation of the mean EE % in terms of coded factors (R^2^ = 0.9998) was:

Mean  EE %=90.67−3.83 X1−4.25 X2+2.92 X3−7.25 X1X2+2.92 X1X3−5.25 X2X3

**Figure 4.**
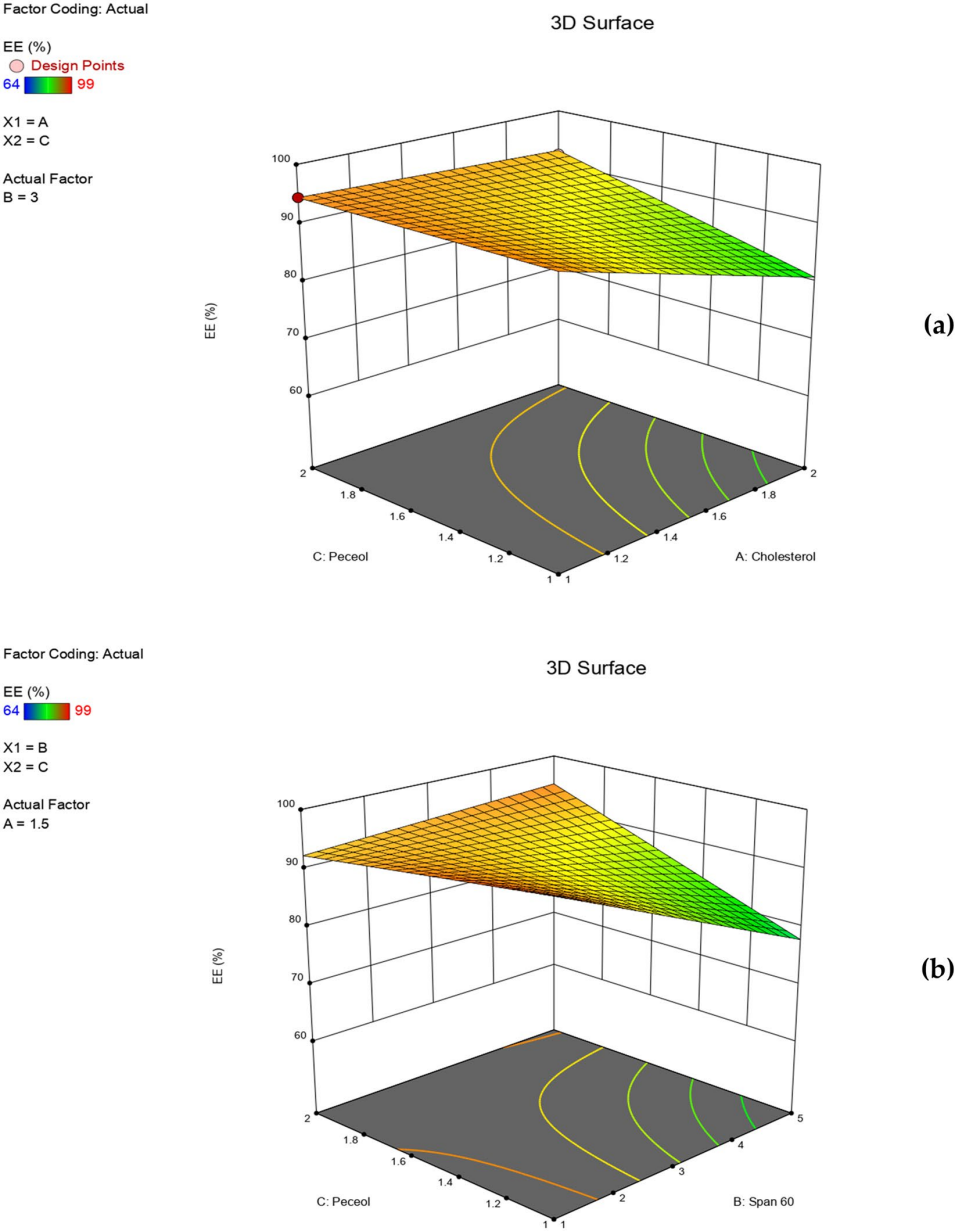
Three dimensional (3D) surface plots for the effect of independent variables on EE %.

In our study, results revealed that EE% was inversely impacted by cholesterol concentration. As clearly shown in Figure 4(a), increased cholesterol concentration is accompanied by a significant decrease in EE%. This could be explained based on the disruption of the regular, linear structure of niosomal membranes upon increasing cholesterol above a certain level. Hence, drug leakage occurs. Also, high cholesterol content could lead to drug-cholesterol competition for the packing space within the niosomal bilayers, therefore excluding the drug from the bilayer structure. These findings correlate well with previous studies on the effect of cholesterol on the EE% of niosomal formulations (Aboelwafa et al., 2010; Thomas & Viswanad, 2012). In addition, increased span 60 concentrations led to decreased EE% values (Figure 4b). This could be due to the formation of mixed micelles, together with niosomal vesicles. Moreover, the decreased EE% due to X_2_X_3_ (Figure 4b), may be due to the increased fluidity of the membrane bilayer by the effect of both span 60 and peceol™ (AbuElfadl et al., 2021) thereby, increasing the niosomes permeability and drug leakage.

### 3.4. Optimization of RPG niosomes

**Figure 5.**
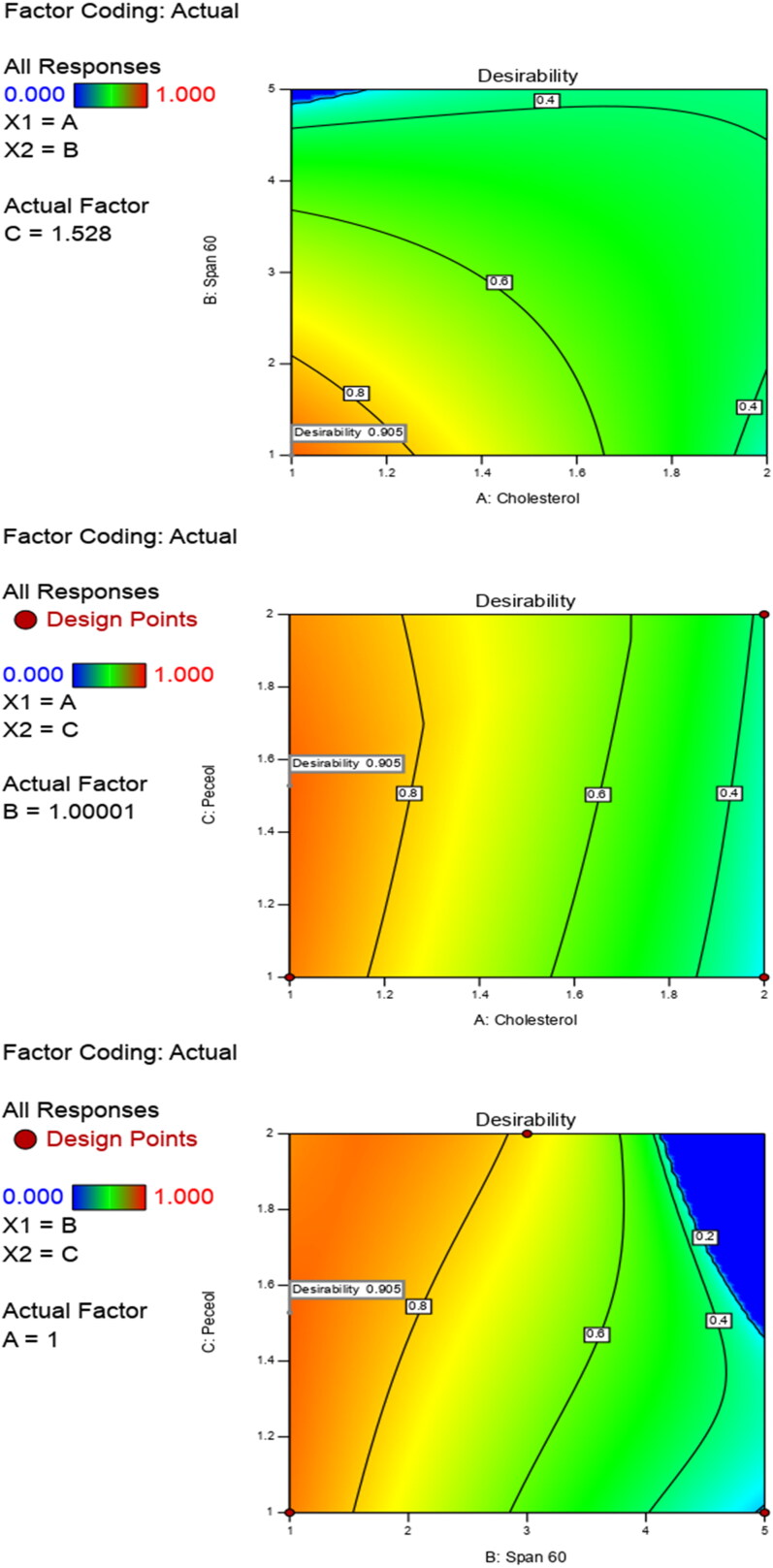
Optimization of independent variables to reach desirability criterion (∼ 1).

Optimum values of independent variables were obtained via the Design^®^ Expert software based on the desirability criterion (Figure 5). The optimized formulation aimed to accomplish the desired characteristics namely; maximized values of EE% and ZP and minimized values of PS and PDI. Therefore, based on the investigated results, the chosen optimized niosomal formulation (ONF) would contain cholesterol, span 60 and peceol^TM^ in the ratio of ‘1:1:1.51’. Results showed great similarity between the observed and the predicted values of the measured responses of ONF, at the maximum desirability (0.99) (Table 3).

**Table 3.** Predicted and observed responses for the optimized RPG niosomal formulation (ONF).

Responses	Predicted	Observed	Residual*
Y_1_: PS (nm)	308.291	306.600 ± 84.00	2.291
Y_2_: ZP (mV)	−39.800	−38.600 ± 1.20	1.200
Y_3_: PDI	0.525	0.480 ± 0.05	0.045
Y_4_: EE (%)	91.192	92.000 ± 2.60	0.808

* Residual = Predicted values – Observed values.

### 3.5. In vitro drug release of ONF

**Figure 6.**
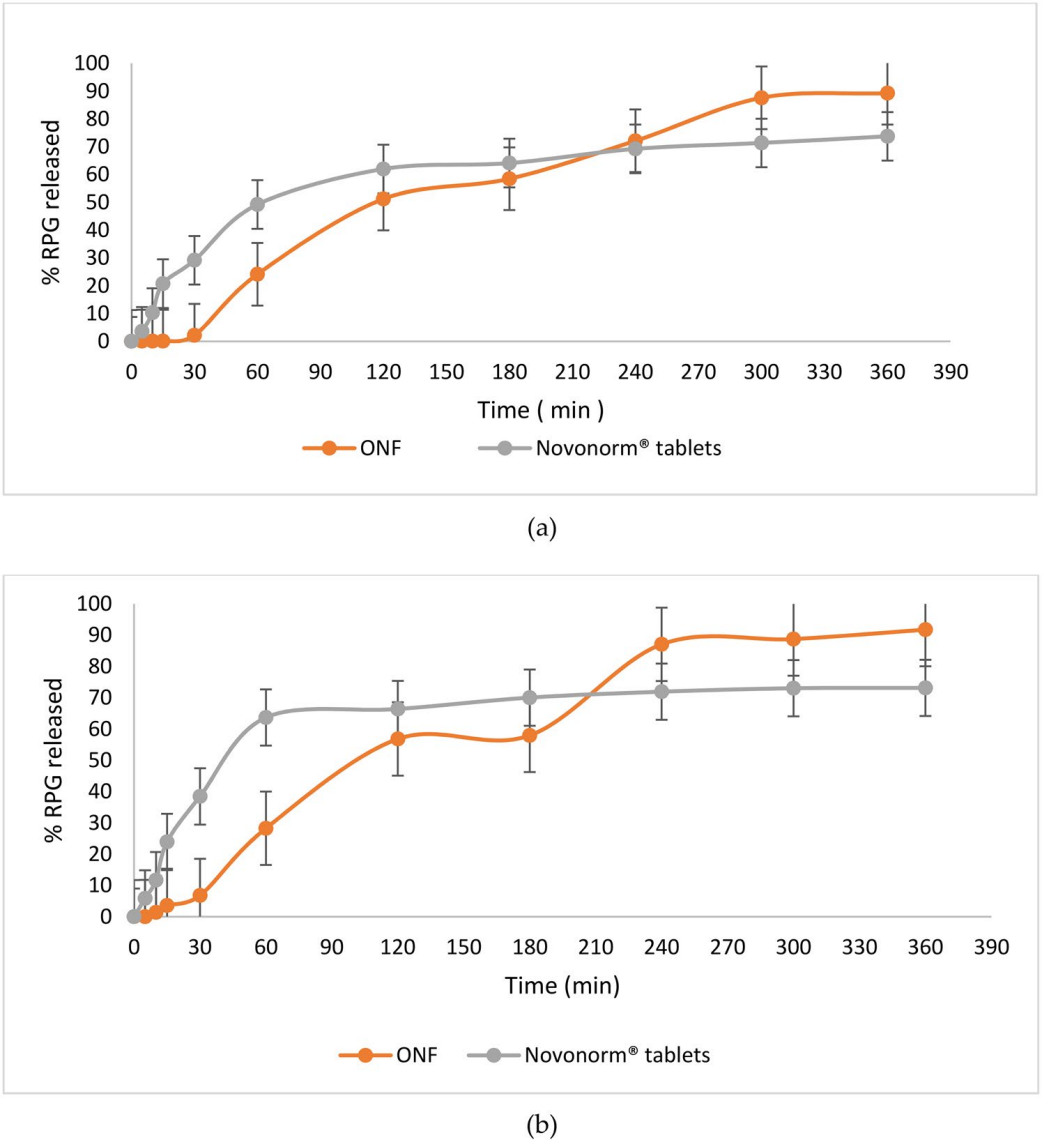
In vitro release profiles of RPG from ONF compared to Novonorm^®^ tablets in SGF (a) and SIF (b). (At 6 h, RPG release from ONF was greater and statistically significantly different (*p* < 0.0001) from Novonorm^®^ tablets, with confidence interval (CI) = 95%).

The in vitro release profiles of RPG from the ONF compared to the oral market product; Novonorm^®^ tablets (2 mg) in two different dissolution media; SGF and SIF are shown in Figure 6 (a and b), respectively. It is clear that RPG release profiles were composed of two phases. ONF showed an initial drug release (> 65%) that lasted for 3.5 h, although lower than that obtained from the oral market tablets, followed by a sustained, but elevated release (compared to Novonorm^®^) that was maintained for 6 h. The initial lowered release behavior could be attributed to the entrapment of RPG within niosomal vesicles. As mentioned in literature, there are many factors that determine the release of entrapped drug from niosomal vesicles, among which is the composition of the lipid bilayer (Devaraj et al., 2002). In our study, ONF is composed of cholesterol, span 60 and peceol^TM^. Generally, niosomes made of cholesterol show an initially lowered release pattern. This is because cholesterol is the sole component responsible for stabilization of the lipid membrane bilayer. RPG, being a lipophilic drug (Ebrahimi et al., 2016) would localize between the fatty acid chains of the lipid bilayer. The lipid bilayer being intact, with reduced permeability and decreased leakage; due to cholesterol, will therefore firstly reduce RPG efflux to the external media during in vitro release studies. Similar results were obtained by Jadon et al. in their study on griseofulvin niosomes (Jadon et al., 2009) and Ramadan et al. in their study on acyclovir niosomes (Ramadan & Singh, 2009). Span 60 was also found to have a role in the initially lowered RPG release from ONF. This is because span 60 is a highly lipophilic surfactant, with high phase transition. Therefore, span 60 molecules follow an ordered gel state at temperature ≥ 25 °C (Attia et al., 2007), which led to an early lowered RPG elution from ONF compared to oral Novonorm^®^ tablets.

The cumulative amount of RPG released from ONF began and continued to increase after 3.5 h, exceeding the oral tablets where it significantly increased by 1.20 and 1.26 fold in SGF and SIF, respectively at 6 h compared to Novonorm^®^ tablets (*p* < 0.0001, in both media). Escalated and extended drug release is attributed to peceol^TM^, the third lipid component (Sachs-Barrable et al., 2007) within the prepared ONF. Peceol^TM^, being an amphiphile (AbuElfadl et al., 2021), acquires high solubilizing power for lipophilic drugs (Kamel et al., 2019). RPG, being lipophilic could attain much enhanced solubility within the lipid bilayer containing peceol^TM^, which led to its consequent dissolution and higher drug concentration in the release media with the gradual erosion of niosomes. Similar results were obtained by Kamel et al. in their study on curcumin (Kamel et al., 2019). It is worth to note that RPG, being a lipophilic drug, led to formation of niosomes having drug-free core and highly drug-rich lipid bilayer, hence being proximal to the dissolution medium which eased RPG release and elution. These results show that ONF can act as a reservoir for enhanced and extended delivery of RPG. Similar results were obtained by Ruckmani et al. in their study on Cytarabine Hydrochloride niosomes (Ruckmani et al., 2000).

### 3.6. Morphology of the optimized niosome formulation

**Figure 7.**
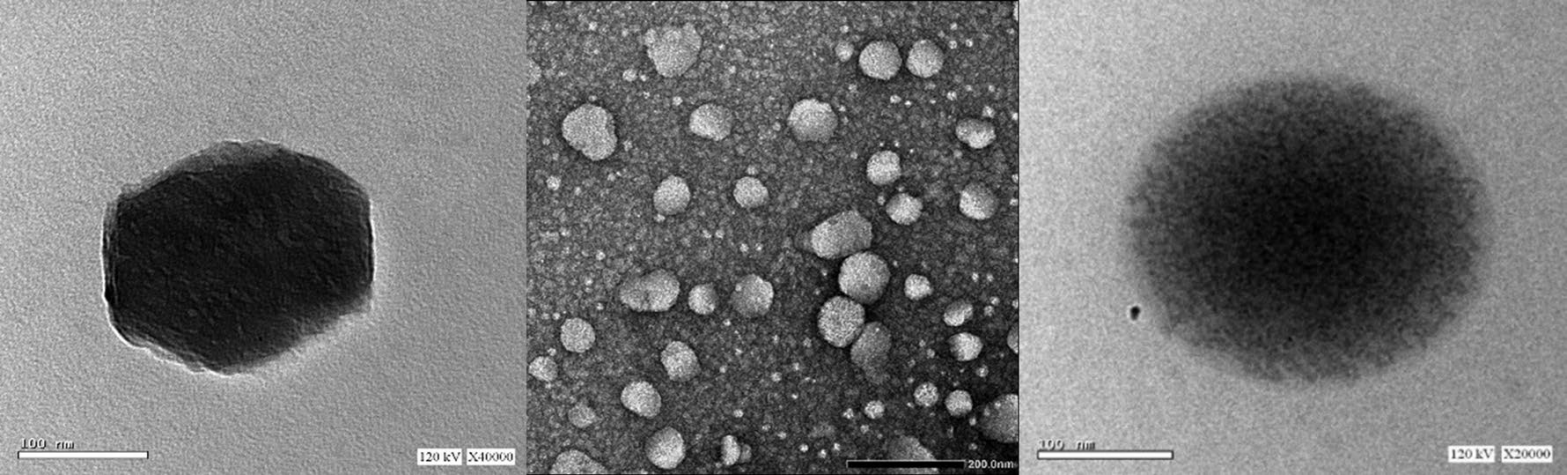
Transmission electron microscope photographs of the ONF.

TEM photographs represented in Figure 7 reveal the morphology of niosomal vesicles of ONF. It is clear that vesicles acquire a spherical shape with a dark core that is surrounded by light grey area representing the bilayer membrane.

### 3.7. Fourier transform infrared spectroscopy (FTIR)

**Figure 8.**
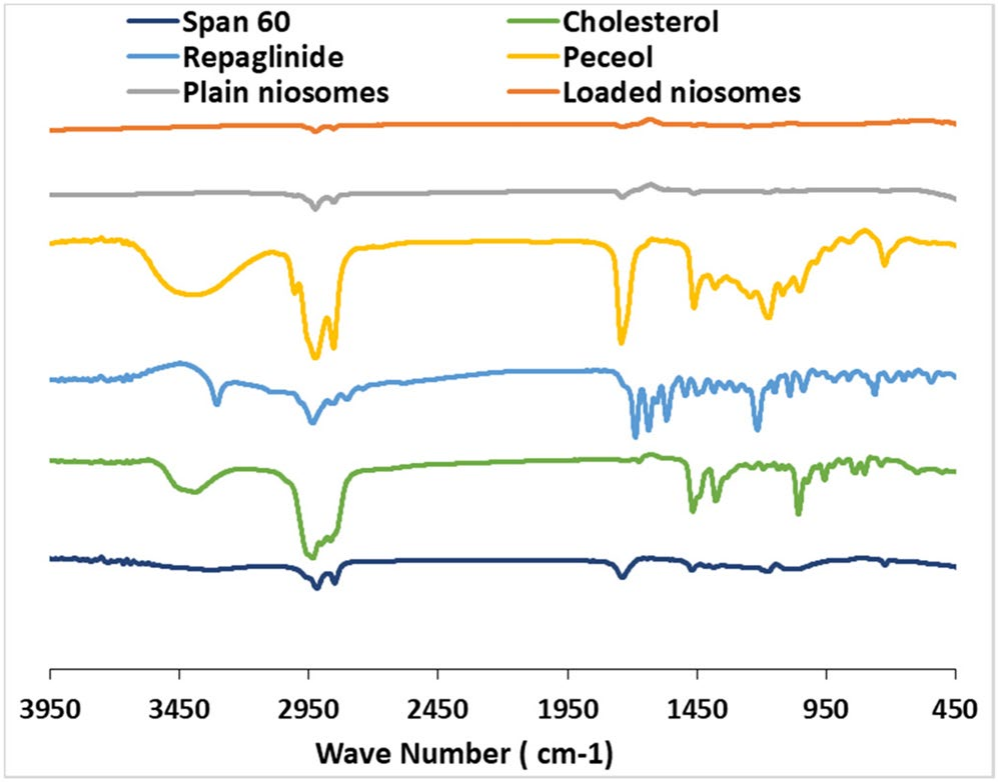
FTIR spectra of repaglinide, cholesterol, span 60, peceol^TM^, ONF (drug loaded optimized niosomal formulation) and plain ONF.

FTIR spectra of RPG, cholesterol, span 60, peceol^TM^, ONF as well as drug-free ONF were recorded (Figure 8). FTIR spectrum of pure RPG showed its characteristic bands at 1690 cm^ − 1^ representing C = O stretching in the carboxylic acid group, 2936 cm^ − 1^ corresponding to CH stretching and 3307 cm^ − 1^ representing NH stretching. Cholesterol FTIR spectrum showed principle peaks at 1055 cm^−1^ representing C = O bending vibration, 1375 cm^−1^ representing C=H bond bending vibration, 2850-3000 cm^−1^ representing CH_2_, CH_3_ group stretching and a broad peak at 3300-3600 cm^−1^ representing = OH group stretching. Characteristic peaks of span 60 appeared at 1738 cm^−1^ representing C = O stretching, 1177 cm^−1^ representing = C= CO = O =, 2916 cm^−1^ and 2850 cm^−1^ representing symmetric and asymmetric aliphatic CH stretching and 721 cm^−1^ for aliphatic CH_2_ rocking. Peceol^TM^ characteristic peak showed 2855 cm^−1^ attributed to the stretching vibration of CH_2_ groups (Wei et al., 2021), and 1,737.96 cm^−1^ corresponding to the C = O valence vibration (Jia et al., 2015). FTIR spectra of ONF was nearly the same as drug-free ONF. In addition, RPG characteristic peaks disappeared within the ONF indicating drug entrapment within niosomal vesicles. Similar results were achieved by Mohamed et al. in their study on vancomycin niosomes (Mohamed et al., 2017).

### 3.8. Evaluation of RPG-loaded chewable tablets

#### 3.8.1. Physical characterization

Direct compression of the ONF with coprocessed excipients resulted in elegant RPG-loaded tablets. All physical characterization tests were within the pharmacopeia limit. All tablets showed friability < 1% (Table 4). Friability values < 1% indicated good mechanical strength, as well as high tolerance to physical handling (Prajapati et al., 2012). Hardness values ranged from 3.9 ± 0.423 Kg to 4.7 ± 0.410 Kg. None of the tablets showed hardness less than 3 kg. According to literature, tablets’ hardness is preferred to range between 2 – 8 Kg (Moqbel et al., 2016). Average thickness of the prepared tablets ranged from 4.1 ± 0.045 mm to 4.4 ± 0.017 mm. All tablets were within the acceptable weight variation range according to the European Pharmacopeia (EP) (Pharmacopoeia, 2002) (Table 4).

**Table 4.** Physical characterization results of RPG-loaded tablets.

RPG Tablets	Friability (%)	Hardness (kg)	Thickness (mm)	Weight (mg)
Pharmaburst^®^ 500	0.78	3.9 ± 0.423	4.1 ± 0.045	187.6 ± 0.012
F-melt^®^	0.50	4.2 ± 0.343	4.2 ± 0.022	186.4 ± 0.023
Prosolv^®^ ODT	0.20	4.7 ± 0.410	4.4 ± 0.017	185.2 ± 0.345

Data are mean values (*n* = 3 ± S.D.).

#### 3.8.2. In vitro release

**Figure 9.**
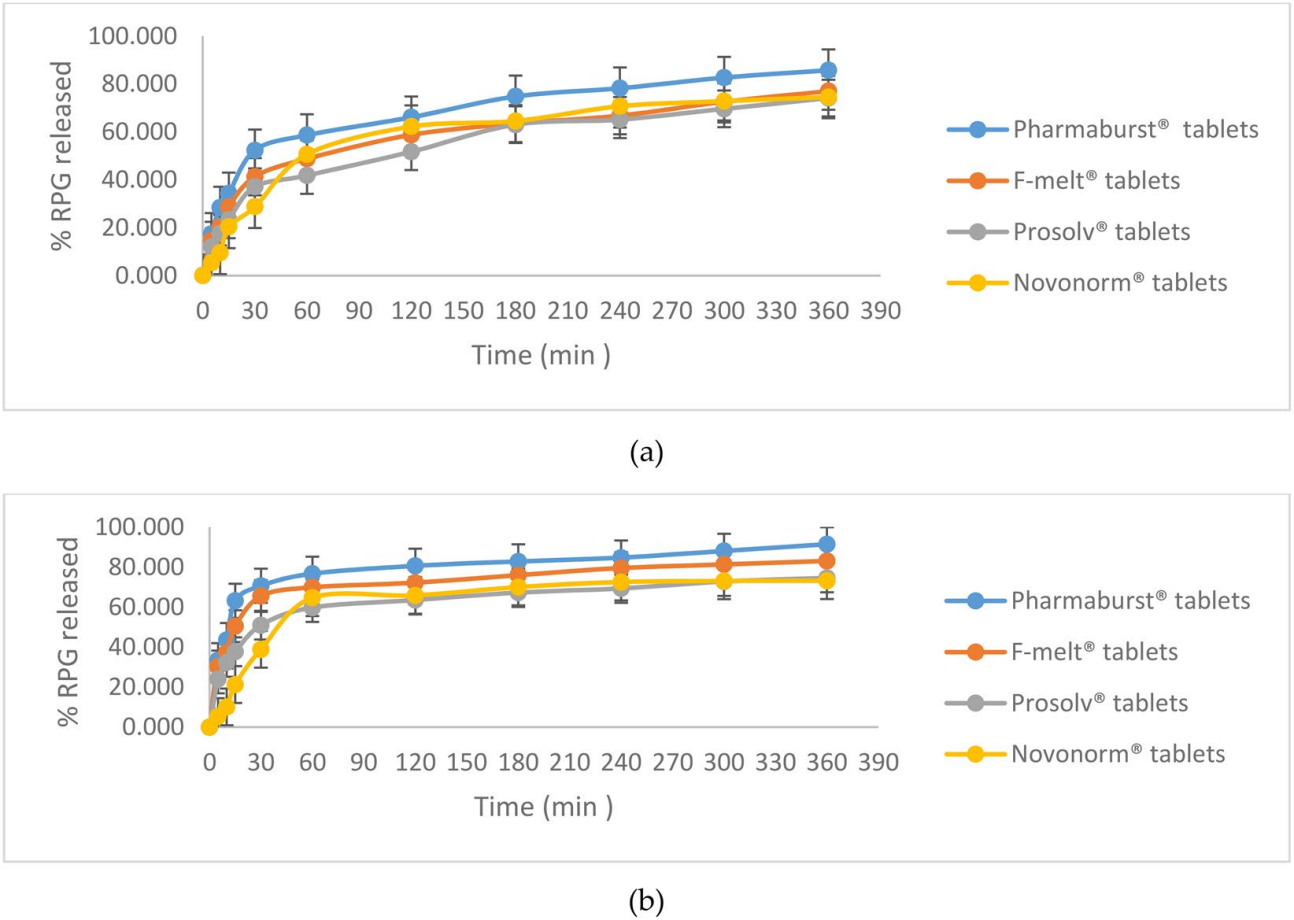
In vitro release profiles of Pharmaburst^®^ 500, F-melt^®^, Prosolv^®^ ODT and Novonorm^®^ tablets in SGF (a) and SIF (b).

RPG chewable tablets showed higher dissolution profiles than Novonorm^®^ tablets in both media; SGF and SIF as shown in Figure 9 (a and b), respectively. As previously mentioned, RPG tablets were formulated using the ONF. An initial robust drug dissolution was shown with all the formulated RPG tablets compared to Novonorm^®^ tablets. At 30 min, dissolution profiles can be arranged in the following descending order Pharmabusrt^®^ 500 > F-melt^®^ > Prosolv^®^ ODT > Novonorm^®^ tablets in both media. In our study, the initial drug burst was realized due to the inclusion of hydrophilic components in coprocessed excipients. All the employed coprocessed excipients namely; Pharmaburst^®^ 500, F-melt^®^ and Prosolv^®^ ODT are mannitol-founded. Mannitol is a hydrophilic ingredient that prompted hastened wetting and solubilization of RPG and hence, enhanced initial drug release. In vitro release profiles, for both media, also showed that Pharmaburst^®^ 500 exhibited the highest percentage of drug released among the three employed coprocessed excipients. This is because Pharmaburst^®^ 500 contains additional sorbitol. Sorbitol is a pore forming agent that can enhance drug release (Sahoo et al., 2017). It fosters porosity within tablets upon contacting the dissolution medium resulting in faster wetting and dissolution. Moreover, it contains crospovidone; a superdisintegrant that conducts hastened absorption of fluids into the tablet via capillarity, and consequently results in rapid and enhanced dissolution. Also, F-melt^®^ tablets exhibited a 1.20 fold and a 1.25 fold higher dissolution than Prosolv^®^ ODT tablets at 30 min in SGF and SIF, respectively. This is because F-melt^®^ contains anhydrous dibasic calcium phosphate which provides enhanced dissolution. Moreover, F-melt^®^ type C, acquires high water absorption ratio with developed porous matrix resulting in enhanced wetting (Krupa et al., 2012). In addition, the presence of crospovidone; the superdisintegrant with dibasic calcium phosphate enhances the wetting of tablets (Fouad et al., 2020) and hence; their dissolution. On the other hand, tablets containing Prosolv^®^ ODT showed the least percentage of drug released although, more than Novonorm^®^ tablets at 30 min, in both media. This is because Prosolv^®^ ODT contains microcrystalline cellulose (MCC). MCC leads to the formation of a hard core matrix which lowers the porosity of tablets; thereby reduces dissolution, fluid uptake and drug release, compared to Pharmaburst^®^ 500 and F-melt^®^ tablets. Similar results were obtained by Fouad et al. in their study on diacerein tablets (Fouad et al., 2020).

Dissolution profiles also showed extended release of RPG in the following descending order; Pharmabusrt^®^ 500, F-melt^®^, Prosolv^®^ ODT and Novonorm^®^ tablets after 6h, in both media. Data were compared using the un-paired t-test, while calculating a 95% CI of the ratio of test to reference tablets. Results showed that Pharmabusrt^®^ 500 exhibited a 1.20 fold and a 1.22 fold statistically significant increase in RPG released after 6h compared to Novonorm^®^ tablets in SGF and SIF, respectively (*p* < 0.0001). Also, F-melt^®^ exhibited a 1.00 fold and a 1.12 fold statistically significant increase in RPG released after 6h compared to Novonorm^®^ tablets in SGF (*p* = 0.0008) and SIF (*p* = 0.0003), respectively. Extended dissolution profiles were due to the sustained effect of ONF previously achieved. However, results showed that Prosolv^®^ ODT tablets acquired an equivalent % of RPG released at 6 h compared to Novonorm^®^ tablets, in both media; SGF and SIF with *p* = 0.3042 and *p* = 0.1279, respectively. Hence, Pharmabusrt^®^ 500 and F-melt^®^ tablets were selected for further in vivo studies.

#### 3.8.3. In vivo study

**Figure 10.**
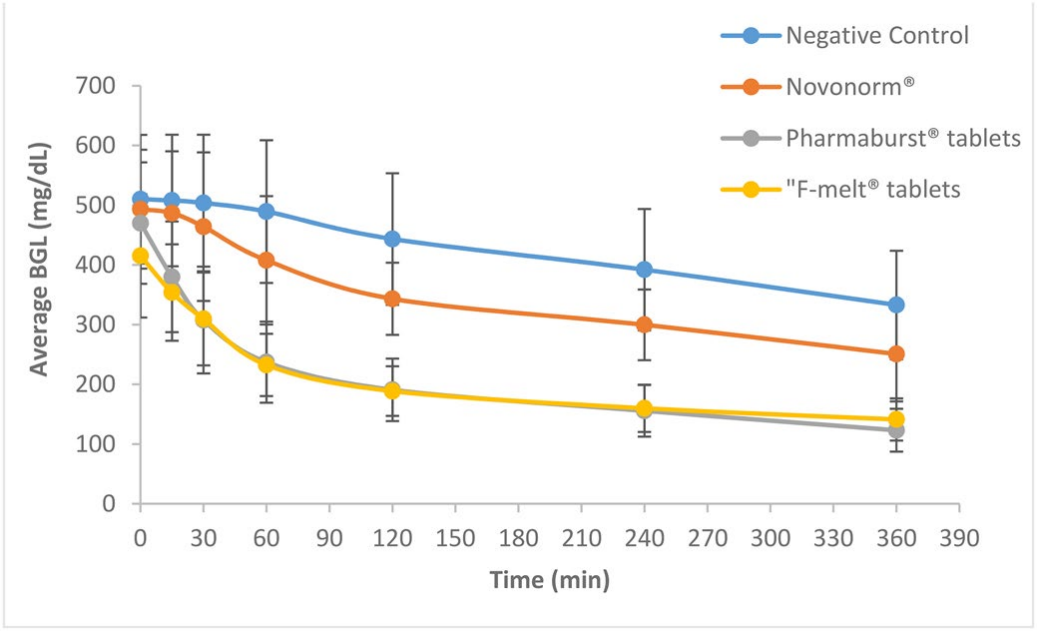
Blood glucose levels of RPG after oral administration of Pharmaburst^®^ 500, F-melt^®^, Prosolv^®^ ODT tablets compared to the market product Novonorm^®^ tablets to diabetic rats (Data are mean values of six determinations ± SEM).

**Figure 11.**
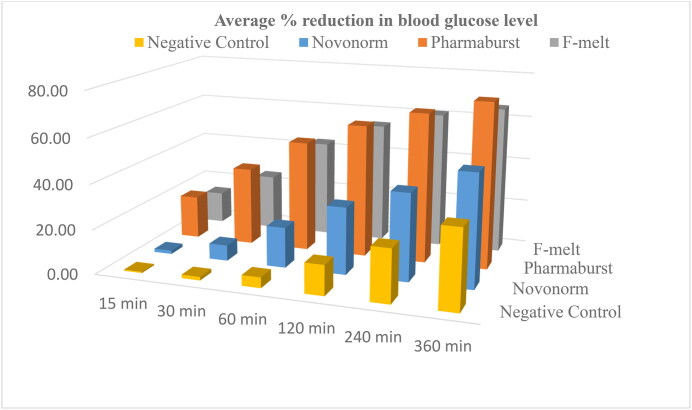
Graphical presentation of % reduction in blood glucose level as a function of time after oral administration of Pharmaburst^®^ 500 and F-melt^®^ tablets compared to the market product Novonorm^®^ tablets to diabetic rats.

The average % reduction in BGL of diabetic rats following oral administration of Pharmaburst^®^ 500, F-melt^®^ and Novonorm^®^ tablets is shown in Figure 10 and graphically represented in Figure 11. A rapid hypoglycemic effect was denoted with a 35.39% ± 6.15 and 25.15% ± 9.64 reduction in blood glucose at 30 min after oral administration of Pharmaburst^®^ 500 and F-melt^®^ tablets, respectively. ANOVA results revealed a 5-fold and a 3.5 fold significantly increased % reduction of blood glucose compared to Novonorm^®^ tablets (*p* < 0.05). These results correlate well with the in vitro release studies performed on the formulated RPG chewable tablets compared to Novonorm^®^ tablets (section 2.9.6.2.). Incorporation of coprocessed excipients proposed rapid absorption of RPG and consequently a hastened fall in blood glucose. Also, rapidly released RPG could have been partly absorbed through the oral cavity membranes and hence, reduced hepatic first pass metabolism (Moqbel et al., 2016). This consequently resulted in rapid RPG absorption and fast hypoglycemic action.

In addition, the hypoglycemic effect was extended through the whole duration of the study to reach a significant increase in % reduction of blood glucose at 6h 74.03% ± 3.36 and 65.95% ± 2.21 for Pharmaburst^®^ 500 and F-melt^®^ tablets, respectively. Both tablets showed a 1.5 fold and a 1.3 fold significantly increased and extended % reduction of blood glucose compared to Novonorm^®^ tablets (*p* < 0.05). These results are strongly correlated with in vitro release profiles of ONF [Figure 6 (a and b)] where, the hypoglycemic effect became sluggish and sustained over a duration of 6h. The extended RPG effect was achieved via the ONF loaded within the formulated chewable tablets. This resulted in drug encapsulation within the lipid bilayer, as well as highly achieved penetration properties of encapsulated RPG through biological membranes (Moqbel et al., 2016; Akhilesh et al., 2011). Previously obtained maximized EE% of the ONF (92.00% ± 2.60) led to sustained drug release of both Pharmaburst^®^ 500 and F-melt^®^ chewable tablets. Additionally, enhanced RPG absorption could be owed to peceol^TM^ via two characteristic mechanisms. Peceol^TM^ plays an important role in avoiding first pass effect due to its role in lymphatic transport (AbuElfadl et al., 2021). In addition, peceol^TM^ enhanced RPG absorption via being a P-group drug efflux pump inhibitor. Similar results were achieved by Gagliardi et al. in their study on doxorubicin hydrochloride (Gagliardi et al., 2020) and Abuelfadl et al. in their study on candesartan cilexetil (AbuElfadl et al., 2021).

## 4. Conclusion

RPG was successfully encapsulated within the prepared niosomes. The ONF was successfully loaded into tablets by direct compression, using three different coprocessed excipients. The resulted in vitro release profiles indicated the enhanced and extended RPG action of the developed ONF, compared to Novonorm^®^ tablets. The in vivo studies reported the significantly improved and sustained hypoglycemic activity of both Pharmaburst^®^ 500 and F-melt^®^ tablets compared to the market product; Novonorm^®^ tablets. Therefore, chewable tablets prepared with coprocessed excipients loaded with ONF could be a promising oral drug delivery system for DM (type II), with enhanced oral absorption properties, especially for patients suffering from dysphagia. They possess both early hypoglycemic effect, as well as extended anti-diabetic activity. However, due to the small number of recruited animals, results can be seen as preliminary and further studies on human volunteers should be performed to prove clinical efficacy of the developed formulations.
